# A Contribution to the Study of Hernias of the Ovary, of the Fallopian Tube and of the Ovary and Fallopian Tube

**Published:** 1916-03

**Authors:** Aime Paul Heineck

**Affiliations:** Chicago, Ill., Surgeon to the Frances Willard, Jefferson Park and Rhodes Avenue Hospitals, Etc.


### A Contribution to the Study of Hernias of the Ovary, of the Fallopian Tube, and of the Ovary and Fallopian Tube.

BY AIME PAUL HEINECK, M. D., CHICAGO, ILL., SURGEON TO THE FRANCES WILLARD, JEFFERSON PARK AND RHODES AVENUE HOSPITALS, ETC.

Hernia is a widespread disease. In the female, the frequency of external hernias has been and is still underestimated. All the hernias herein considered were external hernias, that is, their outermost overlying saccular covering was skin and each, after reaching a certain stage of development, gave rise to a more or less visible, and palpable, external swellings in the ischiadic, obturator, ventral, femoral, inguinal or other region, depending upon the anatomical location of the hernia.

I wish to formulate some conclusions based upon quite an extensive study of the literature and also upon my clinical experience, concerning that type of external hernias in which the hernial sac content is either the Fallopian tube, the ovary or the Fallopian tube and ovary, alone or in association with some other abdominal viscus or viscera.

In investigating the subject, I soon became convinced that deductions and conclusions to be valuable should be based solely upon the study of cases in which the hernial contents had been demonstrated at the operating, dissecting or post-mortem table.

The escape of the uterine appendages from their normal situation may take place through any of the weak spots or openings of the lower abdominal or abdomino-pelvic cavities. A hernia originating either in the internal or in the external inguinal fossa and escaping above Poupart's ligament, is an inguinal hernia; if it escapes beneath the same ligament, and emerges through the crural canal and the saphenous opening, it is a femoral hernia; if through the obturator canal, an obturator hernia; if along the course of the gluteal or sciatic nerves and vessels, emerging almost always above, very infrequently below the pyriformis muscle, very rarely through the lesser sacro-sciatic foramen, a gluteal hernia; if through an operative scar in the abdominal wall, a post-operative hernia.

Though sanctioned by long usage, the classifying of hernias into congenital and acquired is, at times, misleading.

Some hernias are congenital in the truest sense of the word; they are complete at birth, hernial contents being then present. In most of the so-called congenital hernias, the sac only is existent at birth; in an acquired hernia, the sac is always of post-natal development, and in all but hernias "par glissement" is entirely derived from the parietal peritoneum. Congenital hernial sacs result from the want of closure of peritoneal processes, such as the processus vaginalis peritonei in the male, the canal of Nuck in the female, etc., normally present in the foetus. Congenital[1] hernias may appear at any period of life.

Orifices for the transmission of vessels and ducts are normally present in the muscular and aponeurotic layers of the abdominal walls. An acquired hernia is formed by the gradual or sudden escape through these orifices, pathologically widened, of viscera normally contained within the abdominal cavity; the viscera in their passage through and beyond the abdominal wall create paths of escape for themselves by bulging and pushing forward the parietal peritoneum.

### CONCLUSIONS.

1. The Fallopian tube, the ovary,[2] or the tube and ovary,[2] in part or in their entirety, may be herniated. Degree may vary from a complete descent into a hernial sac, of the tube, ovary, or tube and ovary, to a condition where the herniated viscus or viscera lie just without the internal abdominal ring.

2. The herniated tube, ovary, or tube and ovary, may be the sole content of the hernial sac or there may be present as associated hernial contents one, two or more of the following structures or organs: Meckel's diverticulum, appendix vermiformis, omentum, urinary bladder, small or large intestine, rudimentary or fully developed uterus.[3]

3. Tubal, ovarian, and tubo-ovarian hernias are congenital or acquired, unilateral or bilateral; exist alone or in association with one or more other hernias of the same or of dissimilar anatomical types, of the same or of dissimilar clinical characteristics.

4. These hernias, in a small proportion of cases, co-exist with malformations, underdevelopment or absence of other internal or of some external genitalia.

5. In individuals having a herniated tube, a herniated ovary, or a herniated tube and ovary, pathological states of the other internal genitalia or of some external genitalia may be present: Vaginitis, ovarian cystoma, uterine fibroid, uterine prolapse and other uterine displacements, etc.

6. These hernias may co-exist with pathological states of organs other than the internal or external genitalia: Chronic hydrocephalus, multiple stenosis of intestines, hydronephrosis, etc.; these co-existing pathological states not having any relation of cause or effect to the hernial infirmity.

7. Congenital or acquired hernias of the tube, ovary, or tube and ovary, may develop at any period of life. These hernias have been observed in nulliparae, in primiparae, and in multiparae.[3] No age is exempt. No race is immune. As hernias by their complications shorten life duration, the number of hernia-bearing individuals that reach an advanced age is small as compared to that of the non-herniated.

8. According to their anatomical site, hernias of the uterine appendages are designated as post-operative, ventral, gluteal, sciatic or ischiadic, obturator, femoral and inguinal.

9. Clinically, these hernias are reducible, irreducible, non-inflamed, inflamed, strangulated, or their pedicle may be the seat of torsion.

10. Torsion[4] of the pedicle of a herniated ovary or of a herniated tube and ovary, an accident peculiar to, and not infrequent in, hernias of the uterine appendages, gives the same clinical symptoms and determines the same anatomical changes as are observed in strangulated hernias of the uterine appendages.

11. We were able to collect eight times as many inguinal hernias of the internal genitalia as of all the other anatomical varieties put together.

12. Tubal, ovarian, and tubo-ovarian inguinal hernias are recent, old, or recurrent; are direct, interstitial or intra-parietal, indirect or oblique. If indirect or oblique, they are either complete or incomplete. A few sliding hernias are on record.

13. All the bilateral tubal, ovarian, or tubo-ovarian hernias recorded in the medical literature of the last twenty years are of the inguinal variety. In bilateral hernias, both hernias may or may not show the same degree of development; they may have appeared simultaneously or one may have appeared a shorter or longer time before the other. They may show similar or dissimilar clinical characteristics. When bilateral, one hernia may be irreducible and the other reducible.

14. All the hernias in which the complication "torsion of the pedicle" occurred were irreducible congenital inguinal hernias.

15. All the femoral tubal, ovarian or tubo-ovarian hernias recorded in the medical literature of the last twenty years were of the acquired type and appeared in advanced adult life. "Femoral hernia is essentially a hernia of adult life."

16. Hernias of the uterine appendages, in the absence of anomalies of the non-herniated internal genitalia or of the external genitalia, do not if the herniated adnexa be of normal development, free from disease and reducible prevent conception, interfere with gestation, nor unfavorably influence parturition. Pregnancy can occur previous to, during, and subsequent to, the existence of hernias of this nature.

17. The etiology of hernias of the uterine appendages is that of hernia in general. As main factors should be cited:

(1) All conditions associated with increased mobility of the uterine appendages:

(a) Lengthening of the broad ligaments, consecutive to repeated pregnancies.

(b) Pathological relaxation of the ligaments, due to puerperal subinvolution.

(c) Abnormal length of the broad, ovarian, and infundibulo-pelvic ligaments.

(2) All conditions that tend to increase intra-abdominal pressure:

(a) Sudden increase of the intra-abdominal pressure leads to hernia formation by overcoming the resistance offered by one or another of the weak points of the abdominal wall. Sudden increase of the intra-abdominal pressure may lead to the irruption of a tube, ovary, or tube and ovary in the sac of an old enterocele.

(b) Occupations necessitating repeated muscular efforts associated with increased intra-abdominal tension, as the lifting or pushing of heavy weights, etc.

(c) Physiological or pathological states which distend the abdominal cavity, which stretch the abdominal parietes, and widen the orifices normally present in the muscular and aponeurotic layers of the abdominal wall. Enteroptosis, obesity, abdominal tumors, ascites, pregnancy, etc., can be regarded as predisposing and exciting causes to hernia production.

(3) All conditions which weaken the abdominal wall: A hernia can occur wherever the parietal peritoneum is not sufficiently supported by the transversalis fascia and the other structures of the abdominal wall.

(a) Acute or chronic diseases debilitating the organism, especially such as cause great emaciation.

(b) Obesity weakens the abdominal wall and increases the intra-abdominal pressure. The fat present in the abdominal wall, in the omental, mesenteric, and other peritoneal folds explains why obesity plays such a rôle in hernia development.

(c) Traumatism. Most often the traumatism does not cause the hernia, but only reveals its existence. Among traumatisms must be mentioned abdominal operations and their sequelae. Pathologic adhesions of viscera or omentum to the anterior parietal peritoneum near a hernial opening may act as a predisposing cause.

(d) Enteroceles, epiploceles, and entero-epiploceles.

(e) Feeble development or atrophy of the aponeurosis of the transversalis muscle, and of the conjoined tendon. This factor is an important one in direct inguinal hernia.

18. The herniated organ or organs may be free from all degenerative changes.

19. The herniated organ or organs may be bound to the sac-wall or to each other; may be the seat of congestion, gangrene, hemorrhage, inflammation, suppuration, tuberculosis (primary or secondary), cystic and neoplastic disease (benign or malignant).

20. The herniated organ may be the seat of gestation.

21. The hernial sac and the herniated adnexa may be the seat of an inflammation, suppurative or other in character, which, owing to progression by continuity of surface, has extended upward from the vagina, presenting the following anatomical picture: Vaginitis, endocervicitis, endometritis, salpingitis or pyosalpinx, ovaritis and saccular peritonitis.

22. The hernial sac and the herniated contents may be the seat of an inflammation, suppurative or other in character, which, originating in the vagina or in the uterus, has reached the tube and ovary by way of the parametrial and parasalpingeal connective tissue.

23. Pathological processes originating in the hernial contents may, owing to extension by contiguity of tissue, involve the sac and its overlying tissues.

24. Pathological processes, primarily involving the sac or the overlying tissues, can spread to the hernial contents.

25. The hernial sac and the herniated tube, ovary or tube and ovary, can become the seat of an inflammatory or other pathological process originating in the associated hernial contents, epiploitis, appendicitis, gangrenous gut, etc., infection spreading by contiguity of surfaces.

26. The herniated tube, ovary, or tube and ovary, and the associated hernial contents may be free of disease or the uterine adnexa may be normal and pathological changes be present in the associated hernial contents: appendicitis, gangrenous gut, epiploitis, etc.

27. The associated hernial contents may be normal and the herniated uterine adnexa be the seat of morbid changes.

28. It is at times difficult, at times impossible, to determine whether the anatomical changes present in the herniated organ or organs, developed previous to or subsequent to the displacement of the tube, ovary, or tube and ovary, into the hernial sac.

29. Truss treatment for hernias of the uterine appendages is not curative, is often productive of discomfort, and not infrequently interferes with the nutrition and development of the herniated tube or ovary.

30. Women who suffer from any form of hernia should be carefully watched before, during and after their confinement so as to prevent or rather minimize any undue strain upon weak regions of the abdominal wall. These women, at the close of lactation or towards the end of the first year following their confinement, should, in the absence of contra-indications, be subjected to an operation for radical cure of the hernia.

31. After the second year of life spontaneous cure of hernias of the uterine adnexa is rare and can occur only if the hernial contents are easily reduced and easily kept reduced.

32. In females ten years of age or over all hernias irrespective of anatomical site, clinical condition, or nature of contents should, in the absence of a constitutional state contra-indicating operations of election, be subjected to an operation for radical cure.

33. We advise that all hernias of the uterine appendages,[5] whatever be the age of the patient, and irrespective of anatomical site or size, be subjected to an operation for radical cure:

(a) If the hernia be irreducible.

(b) If the hernia be strangulated.

(c) If the pedicle of the herniated organ or organs be the seat of torsion.[6]

After the age of two years:

(d) If the hernias be bilateral.

(e) If other hernias be co-existent.

(f) If the hernia cannot be painlessly, completely, and permanently kept reduced.

(g) If organs other than the uterine appendages be also present in the same hernial sac.

(h) If the wearing of a hernial truss causes pain or aggravates the symptoms.

(i) If the patient has to be subjected to ether, chloroform or other general surgical anesthesia for the performance of an operation of election, double advantage can be taken of this anesthesia, and an operation for the radical cure of the hernia performed.

(j) If patient is exposed to pregnancy.

34. Clinical conditions so closely simulating hernias of the uterine appendages that a positive diagnosis without operation appears impossible, should be subjected to operative treatment. Only benefit can be derived from adherence to this rule. A diagnosis is established, and a cure is effected.

35. In hernias of the uterine appendages, as in all other hernias, the ideal time for operation is previous to the development of degenerative or other pathological states in the herniated organ or organs, and previous to the occurrence of any of the various complications incident to hernias. Early operations give the most satisfactory results.

36. The mortality of operations for the radical cure of hernias, if performed at an opportune time and by a rapid operator, competently assisted, is practically nil.

37. To be effective, operations for radical cure of hernias must well fulfill two essentials: The suppression of the sac and the strengthening of the wall through which the hernia has escaped.

In all herniotomies, the sac should be incised and the hernial contents examined. In the female the inguinal rings are comparatively small. They can, without inconvenience to the patient, be closed.

38. Important operative points:

(a) Always wear and have the assistants wear rubber gloves.

(b) All ligatures and irremovable buried sutures should be of absorbable material.

(c) In inguinal hernias, always divide the aponeurosis of the external oblique muscle to an extent sufficient to give a good exposure of the inguinal canal and of its contents. In the female, the inguinal canal in its normal state and after an inguinal hernia operation, in its restored state, should, outside of a few arterioles and nerve filaments, contain nothing but the round ligament, a structure much smaller than the spermatic cord. This round ligament comes from the muscular structure of the uterus; it finally becomes lost in the labium majus. In a hernia operation, the round ligament, if not the seat of disease, should never be sacrificed.

(d) Always make a high and careful dissection of the hernial sac from the surrounding tissues, and especially from the round ligament, to which it is often quite intimately adherent.

(e) Always open the sac and determine by direct inspection and palpation the nature and state of the hernial contents.

(f) After reduction or ablation of the hernial contents, the sac is to be transfixed and ligated as high as possible. Sac is then removed flush with the peritoneal cavity. This high and thorough removal of the sac is most important.

(g) Never sacrifice the round ligament; it is harmful to the statics of the uterus. Never transplant the round ligament; it is unnecessary. No drainage. After operation, no truss should be worn; a truss does not support the scar; it weakens it.

39. The normal herniated tube or ovary should never be sacrificed. These organs have an important rôle and in the absence of marked structural impairment should be returned to the abdominal cavity.

40. These organs when herniated should be removed, if they be the seat of:

(a) Unavoidable or actual gangrene.

(b) Benign neoplastic disease.

(c) Malignant neoplastic disease.

(d) Voluminous cyst formation (unilocular or multilocular).

(e) Malformation or incomplete development (hydrosalpinx).

(f) Suppurative inflammation.

(g) Hematoma or interstitial ovarian hemorrhage.

(h) Seat of tubal gestation, previous or subsequent to rupture of foetal sac.

(i) Tuberculosis limited to or extending beyond the herniated organ.

(j) Distortion beyond recognition.

(k) Such pathological changes as prevent function.

41. Until we are better informed as to the frequency and nature of true and false hermaphroditism removed herniated uterine adnexa not having a distinctive structure should be subjected to a microscopical examination. This will avoid mistaking testicular for ovarian tissue and vice versa.

42. In the treatment of strangulated sciatic or gluteal, obturator and femoral hernias of the uterine appendages in which the hernial sac also contains gangrenous gut, a double operation is almost always indicated; a laparotomy for the repair of the intestinal lesions, and a herniotomy for the radical cure of the hernia.

43. The herniated tube, ovary or tube and ovary can be removed through the usual herniotomy incisions. The operative steps for the removal of these herniated organs correspond, short of a laparotomy, to the technique ordinarily used in salpingectomy, ovariectomy and oöphorectomy.

BIBLIOGRAPHY.

[1]Matthey (A. L.) Ueber sog. eingeklemmten Hernien der Adnexe. Beitr. z. klin. Chir. Tübingen, 1913, LXXXIII, 361-368.

[2]Rendu (R.) Hernie étranglée de la trompe et de l'ovaire gauches chez une fillette de deux mois et demi., Lyon Med., 1913, CXXII, 25-27.

[2]Petit de la Villeon—Deux cas de hernie de l'ovaire opérés chez l'enfant. J. d. Med. de Bordeaux, 1913.

[3]Farrar (Lillian K. P.) Hernia of the uterus and both adnexa with report of a case (multipara, V-para). Am. J. Obst., N. Y., 1913, LXVIII, 114-120.

[4]Moschcowitz. Torsion of uterine adnexa in hernia of nurslings. Am. J. Obst., N. Y., 1912, LXV, 539-542.

[4]Moschcowitz. A case of torsion of uterine adnexa in hernia in infants. Am. J. Obst., N. Y., 1912, LXV, 537.

[5]Pakowski & Ségard. Hernie de la trompe et de l'ovaire. Bull. mém. Soc. anat. de Par., 1911, LXXXVI, 615-618.

[6]Nové-Josserand (G.) & Rendu. Sur quatre cas de hernie congénitale de la trompe et de l'ovaire chez la petite fille. Arch. Prov. de Chir. Par., 1913, XXII, 543-546.

